# Anesthetic management of a child with catecholaminergic polymorphic ventricular tachycardia undergoing insertion of implantable cardioverter defibrillator : a case report

**DOI:** 10.1186/s40981-020-00322-x

**Published:** 2020-02-26

**Authors:** Keisuke Omiya, Kazuha Mitsui, Takashi Matsukawa

**Affiliations:** 1 Department of Anesthesiology, Faculty of Medicine, University of Yamanashi, 1110 Shimokato Chuo, Yamanashi, 409-3898 Japan; 2 Surgical Center, University of Yamanashi Hospital, 1110 Shimokato Chuo, Yamanashi, 409-3898 Japan

**Keywords:** Catecholaminergic polymorphic ventricular tachycardia, Implantable cardioverter defibrillator, Epicardial pacing, Pediatric anesthesia, Perioperative stress

## Abstract

**Background:**

Catecholaminergic polymorphic ventricular tachycardia (CPVT) is a fatal cardiac ion channelopathy that causes sudden unexpected death in the young.

**Case presentation:**

The patient was a 3-year-old girl with CPVT. Insertion of an implantable cardioverter defibrillator (ICD) using epicardial pacing was scheduled. After premedication of rectal midazolam was given, general anesthesia was induced with midazolam, fentanyl, and rocuronium, and maintained with midazolam, fentanyl, remifentanil, and rocuronium. The operation was performed without any complications. Dexmedetomidine and fentanyl were continuously infused after the operation until she was extubated in the morning of postoperative day 1. Fatal arrhythmia due to perioperative stress did not occur.

**Conclusions:**

We report the anesthetic management of a child with CPVT who underwent insertion of an ICD. CPVT-induced fatal arrhythmia did not occur perioperatively by carefully avoiding perioperative stress with premedication and post-operative sedation.

## Background

Catecholaminergic polymorphic ventricular tachycardia (CPVT) is a potentially fatal disease characterized with ventricular tachycardias that are adrenergic-dependent [1]. The classic electrocardiographic manifestation of CPVT is bidirectional ventricular tachycardia degenerating into self-limiting ventricular fibrillation [2]. The implantable cardioverter defibrillator (ICD) is one of the treatments. However, there are few reports have described general anesthesia in children with CPVT. In this report, we present perioperative management of a child with CPVT who underwent insertion of an ICD using epicardial pacing. This case report was prepared following the CARE guidelines [3].

## Case presentation

The patient was a 3-year-old girl with a height of 107 cm and a weight of 14 kg. She developed ventricular fibrillation while she was running and received cardiopulmonary resuscitation (CPR). She received three times of electrical defibrillation during CPR, but did not receive epinephrine. Return of spontaneous circulation was achieved with 10 min of CPR. Afterward, premature ventricular contraction occurred frequently, and an oral β-blocker (Nadolol) was started. Long QT syndrome and Brugada syndrome were negated in the resting electrocardiogram. Additionally, there was no structural heart disease on the echocardiogram. Hence, insertion of an ICD using epicardial pacing under sternotomy was scheduled due to suspicion of CPVT. Her preoperative neurological condition was intact but nervous, and so premedication of rectal midazolam (0.5 mg/kg) was given. An intravenous catheter was placed preoperatively before the patient entered the operating room. Electrocardiography, percutaneous oxygen saturation, and noninvasive arterial blood pressure were used for monitoring, and electrode pads of a defibrillator were attached to the patient in the operating room. After preoxygenation with 6 L/min oxygen for 3 min, general anesthesia of 3 mg of midazolam, 50 μg of fentanyl, 14 mg of rocuronium intravenously was induced rapidly. She was intubated with a cuffed endotracheal tube (4.0 mm internal diameter). Anesthesia was maintained with a gas mixture of oxygen, air (fraction of inspired oxygen (FIO_2_), 0.3–0.6), and midazolam (0.2 mg/kg/h); additionally, remifentanil (0.15–0.5 μg/kg/min), rocuronium (7 μg/kg/min), and fentanyl (intermittent injection of total 250 μg) were administered. The operation was performed without any complications. The operation time was 3 h. The amount of bleeding was 72 ml. She remained intubated and was transferred to the intensive care unit (ICU). Dexmedetomidine (0.6 μg/kg/h) and fentanyl (approximately 1 μg/kg/h) were administered in the ICU. Tracheal extubation was performed in the morning of postoperative day (POD) 1. Postoperative pain was controlled by suppositories of acetaminophen. Fatal arrhythmia due to CPVT did not occur in the perioperative period. She was discharged from the hospital on POD 21 with oral Nadolol. No fatal arrhythmia has occurred since discharge.

## Discussion

The pathophysiology of CPVT is as follows: the discharge of catecholamine by sympathetic nerve stimulation induces the release of calcium in ionic form (Ca^2+^), and the intracellular Ca^2+^ concentration of cardiomyocytes increases. Fatal arrhythmia is induced by depolarization resulting from this Ca^2+^ concentration imbalance. CPVT is estimated to occur in 1/10,000 [1]. Syncope is the most frequent symptom, and the median age of symptom onset is 10.8 years [1]. Arrhythmia caused by CPVT is caused by physical activity or emotional stress [1]. Although therapeutics, including β-blockers, have been developed for CPVT, treatment failure events, defined as syncope and/or cardiac arrest during a medication, occur in 25% of CPVT patients [4]. Diagnosis is challenging because patients with CPVT have a normal electrocardiogram and echocardiogram; therefore, an exercise stress test that elicits ventricular arrhythmia (bidirectional or polymorphic ventricular tachycardia) is recommended to establish the diagnosis [5].

There are two types of CPVT: juvenile and adult. The juvenile type is classified into types 1–5 according to abnormal gene expression [1, 6]. Aberrant RyR2 gene expression is found in CPVT type 1, which affects 60% of CPVT patients. Inheritance of CPVT type 1 is autosomal dominant, and the median age of symptom onset is 10 years. CPVT type 2 is associated with an aberrant CASQ2 gene, affecting 1% of CPVT patients; the median age of symptom onset is 7 years. CPVT types 3–5 are extremely rare. An aberrant TECRL gene is present in CPVT type 3, and the median age of symptom onset is 10 years. CPVT type 4 has an aberrant CALM1 gene, and the median age of symptom onset is 4 years of age. Those with CPVT type 5 carry an aberrant TRDN gene, and the median age of symptom onset is 2 years. The adult type affects 30% of CPVT patients and occurs predominantly in females. Furthermore, no sudden death has been reported [1, 6]. The patient in this case, had an aberrant CALM1 gene and was therefore classified as CPVT type 4 on POD 78.

In a child with ventricular tachycardia and ventricular fibrillation with a nonparticular or uncertain past history, electrical cardioversion, epinephrine, and amiodarone are used following the resuscitation algorithm [7]. However, epinephrine induces further fatal arrhythmia [8], and amiodarone is not effective for CPVT patients [9]. Thus, confirming the past and family history is crucial to avoid noneffective resuscitation. In this case, nadolol, a long acting β-blocker, was taken orally as pharmacotherapy. Long acting β-blockers are the first choice due to the catecholamine-reducing effect [10, 11].

Regarding anesthetic management, it is important to avoid emotional stress, which causes fatal arrhythmia. In addition, β-blocker treatment should be continued perioperatively [12]. Premedication is useful to avoid fatal arrhythmia induced by catecholamine from stress [12, 13]. In this case, we administered premedication, and she was able to be transferred to the operating room without crying. Insertion of an intravenous catheter is desirable for the infusion of antiarrhythmic agents; however, inhalational induction is an option when insertion of an intravenous catheter is difficult. Highly used anesthetics such as sevoflurane, midazolam, propofol, fentanyl, and remifentanil are available [13, 14]. Ketamine should be avoided because it stimulates sympathetic nervous system activity [13]. General anesthesia may rarely cause severe hypotension in the presence of a large dose β-adrenergic blockade. Positive-pressure ventilation may cause hypotension by reducing venous return. Treatment includes Trendelenburg positioning and appropriate fluid challenge [15]. Administration of a pure α-adrenergic agonist, such as phenylephrine, may be safe in CPVT patients [15]. As β-agonist drugs may induce fatal arrhythmia, hypotension secondary to bradycardia should be treated with atropine or pacing without β-agonist drug use [15]. When arrhythmia occurs, intravenous β-blocker therapy is considered the first choice [10]. Flecainide [1, 10] and verapamil [10, 16] may also be effective. Amiodarone is not effective for CPVT patients [9]. In the present case, phenylephrine, atropine, and landiolol were prepared in preparation for hypotension and arrhythmia.

Perioperative stress can cause fatal arrhythmia, and tracheal intubation until POD 1 was planned using sufficient fentanyl to control postoperative pain. Dexmedetomidine and fentanyl were administered in the ICU. Centrally acting sympatholytic drugs such as dexmedetomidine may be useful in adrenergic-dependent syndrome such as CPVT patients [12]. Dexmedetomidine is also useful to provide smooth extubation [17]. Accordingly, dexmedetomidine was administered in the present case. Additionally, there are some reports that epidural anesthesia is effective for ventricular tachycardia [13, 15, 18]. However, an epidural catheter was not inserted in our case. Nonetheless, epidural anesthesia should be considered in anesthetic management.

We successfully implemented anesthetic management of a child with CPVT who was undergoing insertion of an ICD.

## Data Availability

Data sharing is not applicable to this article because no data sets were generated or analyzed during the current study.

## Abbreviations

Ca^2+^: Calcium in ionic form
CPR: Cardiopulmonary resuscitation
CPVT: Catecholaminergic polymorphic ventricular tachycardia
FIO_2_: Fraction of inspired oxygen
ICD: Implantable cardioverter defibrillator
ICU: Intensive care unit
POD: Postoperative day
